# High fibroblast growth factor 23 levels are associated with decreased ferritin levels and increased intravenous iron doses in hemodialysis patients

**DOI:** 10.1371/journal.pone.0176984

**Published:** 2017-05-05

**Authors:** Hirokazu Honda, Tetsuo Michihata, Kanji Shishido, Keiko Takahashi, Go Takahashi, Nozomu Hosaka, Misa Ikeda, Daisuke Sanada, Takanori Shibata

**Affiliations:** 1 Showa University Koto Toyosu Hospital, Division of Nephrology, Department of Medicine, Tokyo, Japan; 2 Showa University, School of Medicine, Division of Nephrology, Department of Medicine, Tokyo, Japan; 3 Ebara Clinic, Department of Dialysis, Tokyo, Japan; 4 Kawasaki Clinic, Department of Dialysis, Kawasaki, Japan; 5 Division of Dialysis, Kitami Higashiyama Clinic, Tokyo, Japan; 6 Showa University Northern Yokohama Hospital, Department of Internal Medicine, Yokohama, Japan; Hospital Universitario de la Princesa, SPAIN

## Abstract

A recent study demonstrated the association between inflammation, iron metabolism and fibroblast growth factor (FGF) 23. The present clinical study aimed to assess associations between anemia, iron metabolism and FGF23 in hemodialysis (HD) patients. This prospective observational study examined a cohort of prevalent HD patients (n = 282). Blood samples were obtained before dialysis sessions to measure baseline levels of hemoglobin (Hb), transferrin saturation (TSAT), ferritin, albumin-adjusted calcium (Ca), phosphate (P), intact (i)-PTH, 25-hydroxyvitamin D, 1,25-dihydroxyvitamin D, intact (i)-FGF23, high sensitive (hs)-CRP, and interleukin-6. After the baseline measurement, study patients were followed-up for 6 months. Biochemical measurements were subsequently performed at 1 (Hb), 2 (TSAT and ferritin) or 3 (Ca, P and hs-CRP) month intervals. Doses of ESAs and intravenous iron supplementation during the study period were recorded. i-FGF23 was positively correlated with Ca, P, i-PTH and inversely correlated with TSAT and ferritin. However, levels of Hb and hs-CRP and doses of ESAs during the study period did not differ among the i-FGF23 tertiles, with levels of ferritin and TSAT in the higher i-FGF23 tertile being consistently lower than in the middle to lower i-FGF23 tertiles. Multivariate repeated measures analysis indicated that the higher i-FGF23 tertile was independently associated with repeated measurements of ferritin, but not of TSAT. Doses of intravenous iron supplementation were significantly increased in the higher i-FGF23 tertile in multivariate models. In conclusion, high i-FGF23 levels may be associated with prolongation of low levels of ferritin, resulting in increased usages of iron supplementation in HD patients.

## Introduction

Fibroblast growth factor 23 (FGF23) is secreted by osteocytes and is involved in the regulation of phosphorus and vitamin D homeostasis. Excess FGF23 production in individuals with normal kidney function causes hypophosphatemia, low serum parathyroid hormone (PTH) levels, low plasma 1,25 dihydroxyvitamin D_3_ (1,25OHD) levels, and bone demineralization [1]. FGF23 levels are increased in patients with chronic kidney disease (CKD), particularly those on dialysis, in response to increased phosphate levels, increased PTH levels, and intervention of vitamin D [1]. Moreover, high FGF23 levels are associated with increased risk for mortality, cardiovascular disease, hospitalization and heart failure in those patients [2–8]. However, FGF23 regulation is not fully understood in local mineralization during bone turnover and systemic mineral metabolism.

Increased FGF23 levels may be associated with hematopoiesis [9]. A recent study using an autosomal dominant hypophosphatemic rickets model demonstrated that iron deficiency stimulated transcription of FGF23 and increased FGF23 cleavage in osteocytes [9]. While the resultant fragments of FGF23 (namely c-terminal (c-) FGF23) were increased, bioactive, intact (i)-FGF23 remained at stable levels [10]. Another study using an animal model of functional iron deficiency due to chronic inflammation confirmed that inflammation and iron deficiency caused an increased FGF23 production and impaired FGF23 cleavage [11]. In this study, i-FGF23 and c-FGF23 were increased in the CKD model; however, while c-FGF23 was significantly increased, i-FGF23 was reported to be at near normal levels in a normal kidney model [11]. In clinical studies, an inverse association between serum levels of iron and FGF23 has been demonstrated [12–14]. Randomized studies of ferric citrate, a form of iron that includes a phosphate binder, administration causes increased levels of hemoglobin and decreased both phosphate and FGF23 [15–16]. The reduction of c-FGF23 and i-FGF23 appears to be associated with iron levels rather than phosphate levels, because the degree of FGF23 reduction induced by other phosphate binders is not similar. Block GA *et al*. reported that calcium acetate and lanthanum did not reduce FGF23, and sevelamer carbonate produced only a weak reduction in i-FGF23 compared with iron/phosphate binder administration, although these phosphate binders reduced serum phosphate levels to the same degree as iron/phosphate binder administration [17]. While several studies demonstrated that lanthanum could also decrease FGF23 levels [18,19], oral iron supplementation provides the benefit of decreasing FGF23 levels.

On the other hand, results from studies of intravenous ferrotherapy were contrary to oral supplementation, with FGF23 levels reported to increase rather than decrease, although iron status was improved [20,21]. The discrepancy between oral and intravenous iron effects may be associated with decreased intestinal phosphate absorption and other conditions, such as inflammation and oxidative stress, associated with intravenous ferrotherapy. However, the associations among FGF23 levels, iron deficiency and ferrotherapy in CKD patients remain unclear. Thus, the present study aimed to assess the association between intravenous ferrotherapy and iron metabolism, according to i-FGF23 levels in patients undergoing hemodialysis.

## Methods

### Study design and patients

This study was performed as part of an ongoing prospective cohort study that recruited 412 prevalent HD patients treated at Kawasaki Clinic and Ebara Clinic during the period between July 2007 and July 2008. Exclusion criteria were as described in our previous study [22]. Overt gastrointestinal diseases such as untreated gastric cancer and ulcers, as well as intestinal hemorrhage were excluded from the study. The study protocol was approved by the ethics committee of Showa University School of Medicine. All subjects gave informed consent in accordance with the requirements of the institutional committee on human research, and written informed consent was obtained from all patients. The study was performed according to the 2004 revised Helsinki Declaration.

Mineral bone disorders were managed similarly in all patients according to the Japanese Society of Dialysis Therapy Treatment Guidelines [23]. Nutritional management and dialysis protocols were described in detail previously [22].

The study was designed as a prospective observational study with six-months of follow-up after baseline analysis. Of the 412 patients initially enrolled in the study, patients were excluded from the study when stored frozen blood samples were not available for i-FGF23 assay. A power calculation was not used for sample size calculation. Thus, 296 patients were recruited for the present study. Background information and laboratory data between patients with and without FGF23 assessments are shown in S1 Table.

During the follow-up period, 14 patients were withdrawn from the study due to death (n = 3), transfer to another hospital (n = 3), or because iron metabolism data were not obtained during the follow-up period (n = 8). Hence, 282 patients completed the study.

### Administration of ESA

Intravenous epoetin-β (Eposin; Chugai Pharmaceutical Co. Ltd., Tokyo, Japan) or darbepoetin-α (DA; Nesp; Kyowa Hakko Kirin Co. Ltd., Tokyo, Japan) was used for anemia management [24]. As described detail in our previous study [24], anemia was managed according to the Guidelines for Renal Anemia by the Japanese Society for Dialysis Therapy [25].

### Iron supplementation

Iron supplementation was described detail in previous our study [24]. Total ferrotherapy was restricted to thirteen doses per course, and ferrotherapy was discontinued when hemoglobin levels increased above 10 g/dL and serum TSAT and ferritin levels reached the target values.

### Biochemical methods

Blood samples were obtained before the baseline dialysis session to measure routine biochemical parameters, blood count, transferrin saturation (TSAT), ferritin, albumin-adjusted calcium (Ca), phosphate (P), intact (i)-PTH, 25-hydroxyvitamin D, 1,25-dihydroxyvitamin D, i-FGF23, high sensitive (hs)-CRP, and interleukin (IL)-6. The immunonephelometric method was used to measure high-sensitivity C-reactive protein (hsCRP). 25-hydroxyvitamin D and 1,25-dihydroxyvitamin D were measured by radioimmunoassay. Baseline 25-hydroxyvitamin D, 1,25-dihydroxyvitamin D, IL-6 and i-FGF23 were measured using stored frozen blood samples (-80°C). IL-6 measurement was described in detail previously [22]. i-FGF23 was measured using a commercial ELISA kit (Kainos Laboratories Inc., Tokyo, Japan) with an intra-assay CV of < 2.1% and an inter-assay CV of < 4.0%.

After the baseline measurement, study patients were followed-up for 6 months. Subsequent biochemical measurements were performed at 1 (Hb), 2 (TSAT and ferritin) or 3 (Ca, P and hs-CRP) month intervals.

### Statistical analyses

Data presentation and P values for statistical significance were described in our previous study [22]. Statistical analysis for comparisons between two groups of normally distributed variables and non-normally distributed variables were performed using the Student’s t-test and the Wilcoxon rank sum test, respectively. For nominal variables, χ^2^ test was used for comparisons between two or more groups. Correlations of non-parametric data were calculated by Spearman’s rank correlation test. Associations between i-FGF23 and doses of intravenous ferrotherapy over 24 weeks were assessed by multiple linear regression analysis. Associations between TSAT and ferritin, estimated by 4 time points, and i-FGF23 were assessed using a multivariate repeated measurement analysis approach.

Data were analyzed using JMP Pro version 12.0.1 (SAS Institute, Cary, NC, USA), Stata/MP 13.1 (StataCorp, College Station, TX, USA) and Prism version 5.0c (GraphPad Software, Inc., La Jolla, CA, USA).

## Results

Baseline i-FGF23 levels were positively correlated with Ca, P, i-PTH and inversely correlated with TSAT and ferritin (S2 Table). Hemoglobin levels were inversely associated with ferritin, TSAT, hs-CRP and LL-6, and were not correlated with 25-hydroxyvitamin and log 1,25-dihydroxyvitamin D (S2 Table). Levels of hemoglobin and i-FGF23 in users and non-users of calcium acetate, sevelamer carbonate and vitamin D are shown in (S3 Table).

### Patient characteristics and baseline laboratory data according to FGF23 tertiles

To compare the association between FGF23 and iron metabolism, patients were divided into i-FGF23 tertiles. Patients’ characteristics according to i-FGF23 tertiles at baseline are listed in Table 1. Patients in the higher i-FGF23 tertile were younger, had a greater proportion of males, and a lower prevalence of DM than in the lower i-FGF23 tertile (Table 1). The proportion of active vitamin D users in the higher i-FGF23 tertile was greater than in lower i-FGF23 tertile (Table 1).

**Table 1:** Patients’ characteristics.

	All (n = 282)	i-FGF23 higher tertile (n = 94)	i-FGF23 middle tertile (n = 94)	i-FGF23 lower tertile (n = 94)	p
Baseline i-FGF23 levels (ng/mL)	4,847.2 (5.5, 74,309.9)	22,475.4 (9,822.9, 74,309.9)	4,768.7 (2,104.6, 9,667.1)	1,070.0 (5.5, 2,047.2)	
Age (years)	63 ± 13	59 ± 12	63 ± 13	65 ± 11	0.002
Gender (male, %)	63	67	69	52	0.02
Diabetes mellitus (%)	32	21	31	45	0.002
Cause of CKD (%)					0.01
Chronic glomerulonephritis	39	42	45	30	
Diabetic nephropathy	30	20	27	43	
Nephrosclerosis	12	13	14	10	
Polycystic kidney disease	4	4	1	7	
Other diseases	4	4	3	4	
Unknown	10	16	9	6	
Body mass index (kg/m^2^)	21.3 ± 3.3	21.2 ± 3.0	22.0 ± 3.2	21.3 ± 3.3	0.16
History of CVD (yes, %)	48	45	47	52	0.27
Hemodialysis vintage (months)	143 (6, 489)	141 (18, 489)	130 (6, 430)	165 (6, 489)	0.33
Subjective global assessment (%)	15	12	15	21	0.15
Kt/V	1.5 ± 0.3	1.5 ± 0.2	1.5 ± 0.2	1.5 ± 0.3	0.44
Normalized PCR (g/kg/day)	1.01 ± 0.21	1.05 ± 0.20	1.04 ± 0.18	0.94 ± 0.22	0.0009
Phosphate binder (%)	64	63	66	63	0.89
Ca containing phosphate binder (%)	35	47	32	27	0.01
Sevelamer (%)	41	22	46	53	<0.0001
Active vitamin D_3_ (%)	73	80	74	64	0.04
ESA user (%)	88	80	85	82	0.71
Epoetin (%)	41	38	46	39	
Darbepoetin (%)	46	47	45	48	
ESA resistance index					
Epoetin (IU/kg/Hb)	4.9 ± 4.8	5.5 ± 5.3	4.9 ± 5.0	4.3 ± 3.8	0.82
Darbepoetin (μg/kg/Hb)	0.05 ± 0.03	0.05 ± 0.04	0.05 ± 0.03	0.05 ± 0.04	0.64
Intravenous ferrotherapy (%)	46	53	43	41	0.21
Proton-pomp inhibitor (%)	36	33	41	35	0.45

P value for differences of the variables among intact-fibroblast growth factor 23 (i-FGF23) tertiles. CVD: cardiovascular disease, PCR: protein catabolic rate, ESA: erythropoiesis stimulating agents.

Levels of calcium, phosphate and i-PTH were greater in the higher i-FGF23 tertile compared with the middle and lower i-FGF23 tertiles (Table 2). Ferritin and TSAT were lower in the higher i-FGF23 tertile compared with the middle and lower i-FGF23 tertiles (Table 2).

**Table 2:** Laboratory findings at baseline.

	All (n = 282)	i-FGF23 higher tertile (n = 94)	i-FGF23 middle tertile (n = 94)	i-FGF23 lower tertile (n = 94)	p
Baseline i-FGF23 levels (ng/mL)	4,847.2 (5.5, 74,309.9)	22,475.4 (9,822.9, 74,309.9)	4,768.7 (2,104.6, 9,667.1)	1,070.0 (5.5, 2,047.2)	
RBC count (×10^4^/μL)	335 ± 41^b^	338 ± 41	333 ± 44	334 ± 40	0.29
Hemoglobin (g/dL)	10.2 ± 1.0	10.2 ± 1.0	10.2 ± 0.9	10.2 ± 1.1	0.87
MCV (fL)	96.8 ± 6.9	96.6 ± 7.7	96.8 ± 6.7	97.2 ± 6.3	0.96
MCH (pg)	30.6 ± 2.6	30.3 ± 3.1	30.7 ± 2.5	30.8 ± 2.2	0.73
MCHC (%)	31,6 ± 1.3	31.3 ± 1.5	31.7 ± 1.1	31.6 ± 1.2	0.29
Albumin (g/dL)	3.8 ± 0.4	3.9 ± 0.3	3.8 ± 0.3	3.8 ± 0.4	0.38
Creatinine (mg/dL)	11.7 ± 2.7	11.9 ± 2.5	11.5 ± 3.0	11.7 ± 2.7	0.73
Calcium (mg/dL) ^†^	9.2 ± 0.7	9.6 ± 0.6	9.2 ± 0.7	8.9 ± 0.6	<0.0001
Phosphate (mg/dL)	5.4 ± 1.1	6.1 ± 1.2	5.3 ± 1.0	4.8 ± 0.9	<0.0001
Intact-PTH (pg/mL)	159.0 (4.0–1537.0)	240.5 (17.0–1537.0)	138.5 (4.0–962.0)	120 (6.0–561.0)	<0.0001
25OHD (ng/mL)	25.1 ± 8.7	25.8 ± 9.0	25.0 ± 7.8	24.5 ± 9.3	0.61
1,25OHD (pg/mL)	11.7 ± 7.4	10.9 ± 6.2	12.2 ± 7.8	12.2 ± 8.1	0.75
TSAT (%)	20.4 ± 8.9	18.6 ± 8.7	20.6 ± 9.2	22.0 ± 8.7	0.04
Ferritin (ng/mL)	81.0 (5.3–706.2)	57.8 (5.9–301.9)	91.7 (5.3–382.0)	86.9 (8.2–706.2)	<0.0001
HsCRP (mg/dL)	0.09 (0.011–5.68)	0.09 (0.023–3.33)	0.11 (0.024, 3.27)	0.09 (0.011–5.68)	0.33
Interleukin-6 (pg/mL)	3.87 (0.84–84.0)	3.74 (1.17–84.0)	4.30 (1.17–37.6)	3.55 (0.84–78.7)	0.71

P value for differences of the variables among intact fibroblast growth factor 23 (i-FGF23) tertiles.

†Adjusted for albumin.

a: median (range)

b: mean ± SD. RBC: red blood cell, MCV: mean corpuscular volume, MCH: Mean corpuscular hemoglobin, MCHC: Mean corpuscular hemoglobin concentration, PTH: parathyroid hormone, 25OHD: 25-hydroxyvitamin D, 1,25OHD: 1,25-dihydroxyvitamin D, TSAT: transferrin saturation, hsCRP: high-sensitive C-reactive protein.

### Mean hemoglobin, total doses of ESA and intravenous iron supplementation during the study period

Mean hemoglobin levels and total doses of ESA, epoetin or DA, during the 24-week study period did not differ among i-FGF23 tertiles; however, total doses of intravenous iron supplementation were significantly higher in the higher i-FGF23 tertile (Table 3).

**Table 3:** Mean doses of erythropoiesis stimulating agent and intravenous iron, and mean hemoglobin levels over 24 weeks.

	All (n = 282)	i-FGF23 higher tertile (n = 94)	i-FGF23 middle tertile (n = 94)	i-FGF23 lower tertile (n = 94)	P
Mean Hb levels (g/dL)	10.3 ± 0.8	10.3 ± 0.7	10.3 ± 0.7	10.2 ± 0.9	0.76
Mean weekly doses of ESA					
Epoetin-β (IU)	2133.3 ± 1710.7 (n = 119)	2342.7 ± 2024.3 (n = 40)	1953.7 ± 1668.9 (n = 46)	2220.2 ± 1337.7 (n = 33)	0.54
Darbepoetin-α (μg)	24.6 ± 11.6 (n = 113)	24.5 ± 11.8 (n = 43)	25.9 ± 11.5 (n = 35)	23.6 ± 11.8 (n = 35)	0.56
Mean doses of IV iron (mg)	313 ± 195 (n = 253)	361 ± 209 (n = 87)	296 ± 197 (n = 83)	280 ± 170 (n = 83)	0.01

P value for differences of the variables among intact fibroblast growth factor 23 (i-FGF23) tertiles. Hb: hemoglobin, ESA: erythropoiesis stimulating agent, IV iron: intravenous ferrotherapy

### Association of FGF23 on changes in TSAT and ferritin during the study period

**Fig 1:**
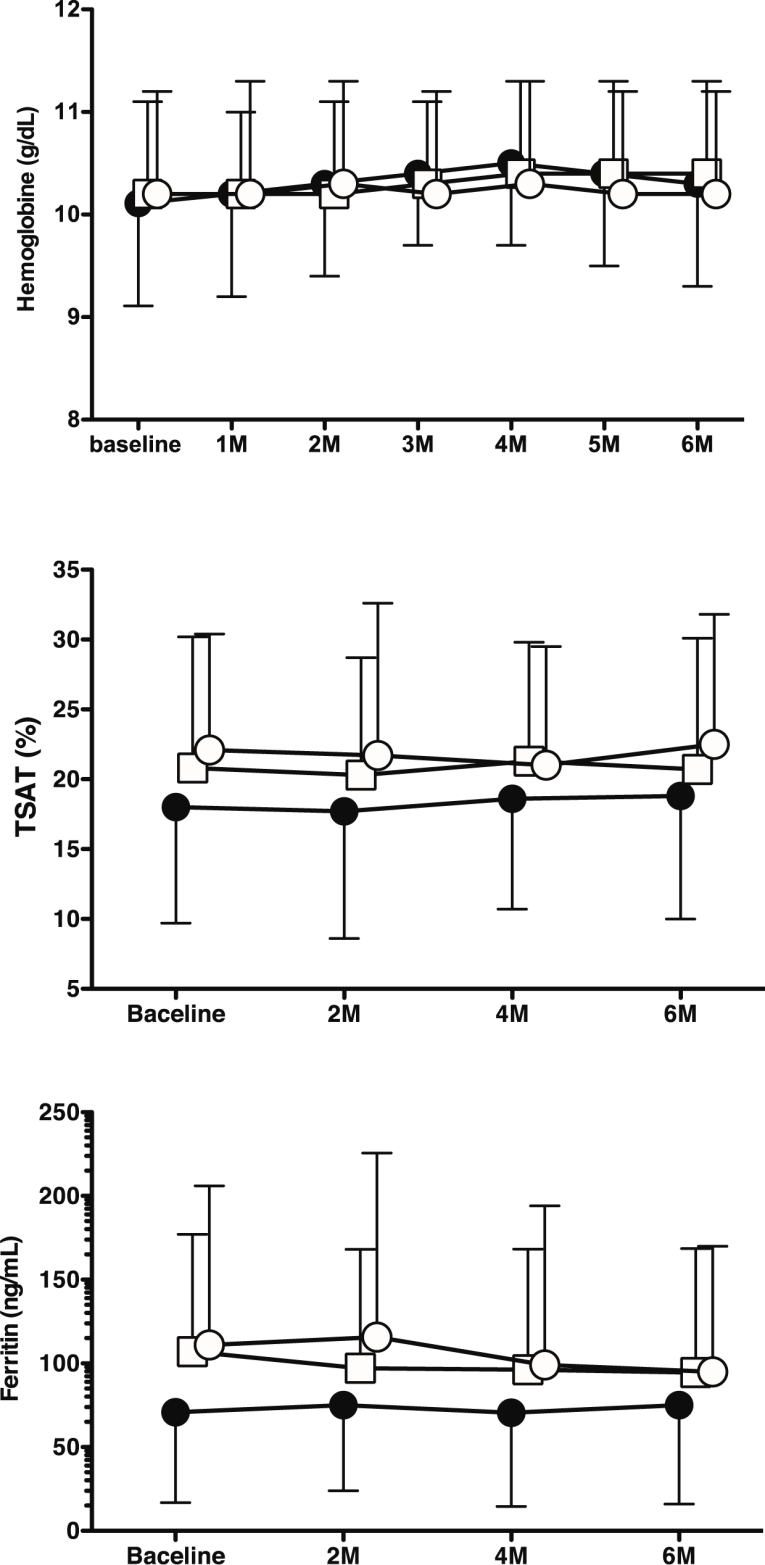
Changes in hemoglobin (a), transferrin saturation (TSAT) (b), and ferritin (c) over 6 months among intact fibroblast growth factor 23 (i-FGF23) tertiles: closed circle, higher i-FGF23 tertile; open square, middle i-FGF23 tertile; open circle, lower i-FGF23 tertile.

Levels of Ca and P were continuously and significantly increased in the higher i-FGF23 tertile (S1 Fig). Whereas levels of Hb and hs-CRP and doses of ESAs during the study period did not differ among the i-FGF23 tertiles (S1 Fig, Fig 1, Table 3), levels of ferritin and TSAT in the higher i-FGF23 tertile were consistently lower than in the middle to lower i-FGF23 tertiles (Fig 1).

The higher i-FGF23 tertile was independently associated with repeated measurements of TSAT and ferritin, according to a univariate repeated measures analysis. However, only the association of ferritin with the higher i-FGF23 tertile, but not TSAT, was confirmed by multivariate repeated measures analysis adjusted with confounders including log hs-CRP, log i-PTH, log 25-hydroxyvitamin D, and log 1,25-dihydroxyvitamin D (S4 Table, Models 1 and 3) and those with treatment of vitamin D, sevelamer and calcium carbonate (S4 Table, Models 2 and 4). Repeated measurements of TSAT were associated with baseline levels of hemoglobin, log ferritin and log hs-CRP.

Doses of intravenous iron supplementation were significantly increased in the higher i-FGF23 tertile compared to the middle to lower i-FGF23 tertiles in the multivariate models (Table 4).

**Table 4:** Association of intact fibroblast growth factor 23 levels with doses of intravenous ferrotherapy over 24 weeks.

	Doses of intravenous ferrotherapy (mg/24 weeks)						
		**Model 1**			**Model 2**		
Values at baseline		**B**	**SE**	**p**	**B**	**SE**	**P**
Higher i-FGF23 tertile		66.2	20.8	0.002	60.9	20.8	0.003
Hemoglobin (g/dL)		-66.2	13.2	<0.0001	-63.3	12.9	<0.0001
TSAT (%)		0.7	1.5	0.61	0.06	1.5	0.98
Log ferritin		-20.0	16.7	0.23	-13.5	16.1	0.40
Log i-PTH		-5.4	15.6	0.73	-11.2	15.6	0.47
Log 25-hydroxyvitamin D		10.3	33.6	0.75	11.2	33.2	0.73
Log 1,25-dihydroxyvitamin D		-7.7	17.9	0.66	-19.6	19.3	0.12
Log hs-CRP		-16.7	10.7	0.12	-17.3	10.4	0.09
		**Model 3**			**Model 4**		
		**B**	**SE**	**P**	**B**	**SE**	**P**
log i-FGF23		25.1	10.0	0.01	22.6	10.3	0.03
Hemoglobin (g/dL)		-66.8	13.2	<0.0001	-64.2	13.0	<0.0001
TSAT (%)		1.0	1.5	0.49	0.4	1.4	0.80
Log ferritin		-24.5	16.7	0.14	-18.8	15.3	0.23
Log i-PTH		-3.2	15.6	0.83	-9.6	15.6	0.53
Log 25-hydroxyvitamin D		9.7	33.8	0.77	9.9	33.4	0.77
Log 1,25-dihydroxyvitamin D		-6.8	18.0	0.70	-30.0	19.4	0.12
Log hs-CRP		-21.0	10.5	0.04	-17.4	10.4	0.09

i-FGF23: intact fibroblast growth factor 23 i-FGF23, TSAT: transferrin saturation, hs-CRP: high sensitive C-reactive protein. Models 1 and 3 were adjusted with age (year), sex (man vs. woman), status of diabetes mellitus (yes vs. no), history of cardiovascular disease (yes vs. no), nutritional state (normal vs. mild to severe malnourished by subjective global assessment), normalized protein catabolic rate (g/kg/day), hemodialysis vintage (months), calcium adjusted for albumin (mg/dL), phosphate (mg/dL). Models 2 and 4 were adjusted with age (year), sex (man vs. woman), status of diabetes mellitus (yes vs. no), hemodialysis vintage (months), calcium adjusted for albumin (mg/dL), phosphate (mg/dL), and treatment of vitamin D and phosphate binder (sevelamer and calcium carbonate).

## Discussion

The present study demonstrated that higher i-FGF23 was associated with consistently lower levels of ferritin and TSAT, and greater doses of intravenous iron supplementation compared to the middle to lower i-FGF23 groups. However, hemoglobin levels and doses of ESA during the observation period did not differ among the i-FGF23 tertiles.

In the present study, ferrotherapy intervention was performed by intravenous iron supplementation with saccharated ferric oxide. Previous studies are consistent with the effect of intravenous iron supplementation on i-FGF23. Roberts *et al*. demonstrated that the effect of ferrotherapy on i-FGF23 may differ between ferric carboxymaltose and iron sucrose, which is in the same category as saccharated ferric oxide [26]. Furthermore, ferric carboxymaltose may inhibit i-FGF23 degradation in osteocytes resulting in transiently increased i-FGF23 levels [21]. This effect was maintained for 2 weeks in female non-CKD patients with a history of heavy uterine bleeding. Takeda *et al*. reported that thrice-weekly administration of saccharated ferric oxide might have caused increased i-FGF23 levels in patients under HD [20]. The effect of ferrotherapy on i-FGF23 was diminished 2 weeks after the discontinuation of iron supplementation. Thus, maintenance ferrotherapy with saccharated ferric oxide may cause increased i-FGF23 levels and the effect of intravenous iron on FGF23 may depend on the type of drug. However, levels of TSAT and ferritin were previously reported to be significantly increased by iron supplementation [20]. These findings were contrary to our observation that TSAT and ferritin were lower in the higher i-FGF23 tertile. While the discrepancy may be associated with the doses of iron supplementation, unknown pathophysiology in patients with higher FGF23 levels may influence iron metabolism because i-FGF23 levels in the higher i-FGF23 tertile were much higher than those reported in Takeda’s study [20].

Another reason may be associated with hepcidin-25 metabolism. While there is no evidence that FGF23 directly moderates hepcidin-25 production levels, a study of CKD models in wild-type and hepcidin knockout mice showed that i-FGF23 levels were increased in both CKD models [27]. However, liver iron levels were increased rather than decreased in those models. This phenomenon was thought to be attributable to the increased absorption of iron from daily oral supplementation in those models due to hepcidin knockout, since liver iron levels were significantly lower in those models when fed low dose iron supplementation compared to standard iron supplementation [27]. In the present study, intravenous iron therapy was performed intermittently and none of the patients were treated orally with daily iron preparations. Thus, differences in iron supplementation may influence storage iron levels. However, we did not describe the detailed relationship between iron metabolism and i-FGF23 because we could not measure hepcidin-25 and biomarkers of iron status such as the percentage of hypochromic erythrocytes. Thus, further research using a well-designed large prospective cohort study is required to examine this relationship.

David *et al*. reported that absolute iron deficiency in 6-week-old mice fed a low iron diet showed increased levels of both c-FGF23 and i-FGF23 [11]. However, functional iron deficiency induced by hepcidin injection in wild-type mice significantly up-regulated FGF23 mRNA and increased serum c-FGF23 revels, while i-FGF23 levels were not elevated in this model [11]. Thus, absolute, but not functional, iron deficiency might increase both c-FGF23 and i-FGF23 levels. It was speculated that these differences were due to age- and growth-dependent differences that might influence FGF23 production and cleavage [11]. However, a clinical study of non-CKD women with absolute iron deficiency due to uterine bleeding showed only c-FGF23 elevation and their i-FGF23 levels were unchanged [20]. Thus, further clinical studies are required to confirm the association between iron metabolism and FGF23.

Elevated FGF23 states may be linked to inflammation. Recent studies in animal models showed an association between inflammation and FGF23; while inflammation increased FGF23 levels via suppression of FGF23 degradation, FGF23 could increase inflammatory cytokine production [11, 28]. Thus, high FGF23 levels may be accompanied by inflammation, resulting in the development of functional iron deficiency. However, our study could not detect a significant inflammatory state and functional iron deficiency in the higher i-FGF23 tertile. Specifically, ferritin levels were lower in the higher i-FGF23 tertile and were higher in the middle to lower i-FGF23 tertiles. Mendoza *et al*. reported that inflammatory biomarkers, such as CRP and IL-6, were independently associated with increases in c-FGF23 levels in a CKD population [29]. However, recent clinical studies reported that these associations were only confirmed in non-CKD patients, and inflammatory biomarkers were not correlated with c-FGF23 levels in CKD patients using a multivariate model [30, 31]. These discrepancies might be associated with the statistical power of different cohort sizes in each study. However, the mechanism of increased FGF23 production and the regulation of i-FGF23 by cleavage to c-FGF23 under chronic inflammation during end-stage kidney disease is not fully understood.

Iron supplementation may induce inflammation via increased oxidative stress [32], and thus may cause elevation of FGF23. In the present study, iron supplementation was performed intermittently when patients showed hemoglobin levels less than the target range accompanied with ferritin levels < 100 pg/mL and TSAT < 20%. Furthermore, iron supplementation was not performed in patients who had hemoglobin levels in the target range yet exhibited ferritin levels < 100 pg/mL and/or TSAT < 20%. Moreover, intravenous iron supplementation was performed once per week in many cases. Thus, rather low doses of iron may not affect the association between inflammation and i-FGF23.

PTH levels are associated with erythropoietic responses in patients under HD [33]. In the present study, i-PTH levels were continuously increased in the high i-FGF23 tertile; thus, high i-PTH may lower ferritin levels. However, i-PTH levels did not affect ferritin levels according to the repeat measures analysis. Moreover, hemoglobin levels and ESA doses did not differ between FGF23 tertiles. Therefore, the impact of i-PTH elevation on anemia was not confirmed in the present study.

The present study results must be viewed with the following caveats. The number of patients was relatively small, the single-point measurement of i-FGF23 precluded accurate baseline evaluations as well as alterations in i-FGF23 throughout the observation period. Moreover, we are not able to determine whether or not ferrotherapy with saccharated ferric oxide might have caused elevated FGF23. Another limitation of the present study was that it excluded the patients who did not complete the analysis; thus, there may be a bias related to patients withdrawing from the study. In the present study, patients were excluded from the study if they exhibited overt gastrointestinal diseases such as untreated cancer and ulcers. However, we were not able to exclude patients with latent gastrointestinal diseases. Furthermore, the findings of this study may have been influenced by unexpected diseases and factors that affect anemia in CKD. Finally, we could not confirm a specific association between FGF23, oral iron supplementation and iron status. A large prospective cohort study that examines the method of iron supplementation is required to clarify the association of FGF23 with the progression of iron deficiency in prevalent HD patients.

## Conclusion

High i-FGF23 levels may be associated with prolongation of low levels of ferritin, resulting in increased usage of iron supplementation in HD patients.

## Supporting information

S1 Fig Changes in Ca adjusted-albumin (Ca_alb_) (a), phosphate (P) (b), and high sensitive (hs)-CRP (c) over 6 months among intact fibroblast growth factor 23 (i-FGF23) tertiles: closed circle, higher i-FGF23 tertile; open square, middle i-FGF23 tertile; open circle, lower i-FGF23 tertile.
(EPS)

S1 Table Background patient information with and without FGF23 measurement. (DOCX)

S2 Table Spearman correlation matrix among biomarkers at baseline. (DOCX)

S3 Table Association of phosphate binders and vitamin D with hemoglobin and biomarkers of iron metabolism and CKD-MBD. (DOCX)

S4 Table Associations of higher i-FGF23 tertiles with repeated measurements of ferritin and TSAT. (DOCX)
