# Correction: High fibroblast growth factor 23 levels are associated with decreased ferritin levels and increased intravenous iron doses in hemodialysis patients

**DOI:** 10.1371/journal.pone.0179139

**Published:** 2017-06-01

**Authors:** The PLOS ONE Staff

The affiliation for the last author is incorrect. Takanori Shibata is not affiliated with #6 but with #2 Showa University, School of Medicine, Division of Nephrology, Department of Medicine, Tokyo, Japan. The publisher apologizes for the error.
