# Supplementary material for: High fibroblast growth factor 23 levels are associated with decreased ferritin levels and increased intravenous iron doses in hemodialysis patients
Source: PLoS One. 2017 May 5;12(5):e0176984. doi: 10.1371/journal.pone.0176984 (PMC5419608; doi:10.1371/journal.pone.0176984)
Supplement: S1 Table — (DOCX) [file pone.0176984.s002.docx]

S1 Table. Backgrounds between patients with and without FGF23 measurement.

|  | FGF23 measured  (n = 282) | FGF23 not measured  (n = 130) | p |
| --- | --- | --- | --- |
| Age (years) | 63 ± 13 | 64 ± 14 | 0.16 |
| Gender (male, %) | 63 | 60 | 0.66 |
| Diabetes mellitus (%) | 32 | 37 | 0.28 |
| Cause of CKD (%) |  |  | 0.12 |
| Chronic glomerulonephritis | 39 | 26 |  |
| Diabetic nephropathy | 30 | 39 |  |
| Nephrosclerosis | 12 | 16 |  |
| Polycystic kidney disease | 4 | 3 |  |
| Other diseases | 4 | 3 |  |
| Unknown | 10 | 12 |  |
| Body mass index (kg/m^2^) | 21.3 ± 3.3 | 21.0 ± 3.5 | 0.14 |
| History of CVD (yes, %) | 48 | 44 | 0.37 |
| Hemodialysis vintage (months) | 143 (6, 489) | 125 (7, 446) | 0.07 |
| Subjective global assessment (%) | 15 | 12 | 0.15 |
| Kt/V | 1.48 ± 0.3 | 1.43 ± 0.2 | 0.06 |
| Normalized PCR (g/kg/day) | 1.01 ± 0.21 | 1.00 ± 0.18 | 0.84 |
| Phosphate binder (%) | 64 | 62 | 0.89 |
| Ca containing phosphate binder (%) | 35 | 29 | 0.18 |
| Sevelamer (%) | 41 | 46 | 0.33 |
| Active vitamin D_3_ (%) | 73 | 64 | 0.06 |
| ESA user (%) | 88 | 82 | 0.74 |
| Intravenous ferrotherapy (%) | 46 | 50 | 0.34 |
| Hemoglobin (g/dL) | 10.2 ± 1.0 | 10.0 ± 0.9 | 0.09 |
| Albumin (g/dL) | 3.8 ± 0.4 | 3.9 ± 0.3 | 0.52 |
| Creatinine (mg/dL) | 11.7 ± 2.7 | 11.1 ± 3.0 | 0.08 |
| Calcium (mg/dL) ^†^ | 9.2 ± 0.7 | 9.3 ± 0.7 | 0.52 |
| Phosphate (mg/dL) | 5.4 ± 1.1 | 5.6 ± 1.2 | 0.22 |
| Intact-PTH (pg/mL) | 159.0 (4.0 – 1537.0) | 150.0 (5.0 - 801.0) | 0.06 |
| TSAT (%) | 20.4 ± 8.9 | 19.7 ± 10.5 | 0.21 |
| Ferritin (ng/mL) | 81.0 (5.3 – 706.2) | 59.0 (6.2 – 662.0) | 0.06 |
| HsCRP (mg/dL) | 0.09 (0.011 – 5.68) | 0.09 (0.005 – 2.70) | 0.45 |

CVD: cardiovascular disease, PCR: protein catabolic rate, ESA: erythropoiesis stimulating agents. †Adjusted for albumin. PTH: parathyroid hormone,TSAT: transferrin saturation, hsCRP: high-sensitive C-reactive protein.
