# Supplementary material for: High fibroblast growth factor 23 levels are associated with decreased ferritin levels and increased intravenous iron doses in hemodialysis patients
Source: PLoS One. 2017 May 5;12(5):e0176984. doi: 10.1371/journal.pone.0176984 (PMC5419608; doi:10.1371/journal.pone.0176984)
Supplement: S2 Table — (DOCX) [file pone.0176984.s003.docx]

S2 Table. Spearman correlation matrix among biomarkers at baseline

|  | **Ca** | **Phosphate** | **i-PTH** | **hemoglobin** | **TSAT** | **ferritin** | **25OHD** | **1,25OHD** | **hs-CRP** | **IL-6** |
| --- | --- | --- | --- | --- | --- | --- | --- | --- | --- | --- |
| **i-FGF23** | 0.46**** | 0.50**** | 0.45**** | -0.001 | -0.18** | -0.20** | 0.06 | -0.05 | 0.03 | 0.005 |
| **Ca** | - | 0.03 | 0.46**** | -0.03 | 0.04 | -0.001 | 0.07 | 0.005 | -0.04 | -0.03 |
| **Phosphate** |  | - | 0.27**** | -0.01 | -0.22*** | -0.19*** | 0.11 | -0.04 | 0.07 | -0.09 |
| **i-PTH** |  |  | - | -0.03 | -0.15* | -0.19** | -0.01 | 0.06 | 0.15* | 0.02 |
| **hemoglobin** |  |  |  | - | 0.18** | -0.20** | -0.01 | -0.01 | -0.16** | -0.18** |
| **TSAT** |  |  |  |  | - | 0.38**** | 0.06 | -0.04 | -0.13* | -0.16** |
| **Ferritin** |  |  |  |  |  | - | 0.03 | 0.001 | 0.01 | 0.10 |
| **25OHD** |  |  |  |  |  |  | - | 0.12* | 0.06 | -0.16* |
| **1,25OHD** |  |  |  |  |  |  |  | - | -0.04 | 0.08 |
| **hs-CRP** |  |  |  |  |  |  |  |  | - | 0.50**** |

*: p<0.05, **: p<0.01, ***: p<0.001, ****: p<0.0001. i-FGF23: intact fibroblast growth factor 23, Ca: calcium adjusted for albumin. i-PTH: intact parathyroid hormone, TSAT: transferrin saturation, 25OHD: 25-hydroxyvitamin D, 1,25OHD: 1,25-dihydroxyvitamin D, hs-CRP: high-sensitive C-reactive protein, IL-6: interleukin-6.
