# Supplementary material for: High fibroblast growth factor 23 levels are associated with decreased ferritin levels and increased intravenous iron doses in hemodialysis patients
Source: PLoS One. 2017 May 5;12(5):e0176984. doi: 10.1371/journal.pone.0176984 (PMC5419608; doi:10.1371/journal.pone.0176984)
Supplement: S3 Table — (DOCX) [file pone.0176984.s004.docx]

S3 Table. Association of phosphate binders and vitamin D with hemoglobin and biomarkers of iron metabolism and CKD-MBD.

|  | Calcium carbonate | | Sevelamer hydrochloric acid | | Vitamin D | |
| --- | --- | --- | --- | --- | --- | --- |
|  | **User** | **Non-user** | **User** | **Non-user** | **User** | **Non-user** |
| i-FGF 23 | 8282.9 (17.9, 90000)* | 3419.0 (5.5, 74310.0) | 2807.3 (35.6, 44697.9) | 8211.7 (5.5, 90000)* | 5707.2 (5.5, 90000)* | 2588.8 (35.6, 74310.0) |
| Calcium | 9.5±0.7* | 9.1±0.7 | 9.1±0.7 | 9.3±0.7* | 9.0±0.8 | 9.3±0.7* |
| Phosphate | 5.4±1.2 | 5.4±1.2 | 5.2±1.3* | 5.5±1.1 | 5.6±1.2 | 5.3±1.2 |
| i-PTH | 217 (38, 954)* | 136.5 (4, 1537) | 149 (6, 1085) | 162.5 (4, 1537) | 128 (4, 1537) | 167 (9, 962)* |
| Hemoglobin | 10.2±0.9 | 10.1±1.0 | 10.2±0.9 | 10.1±1.0 | 10.2±1.0 | 10.2±1.1 |
| TSAT | 21.6±10.0 | 19.8±8.2 | 21.1±8.7 | 19.9±9.1 | 19.8±9.3 | 20.9±8.8 |
| Ferritin | 85.4 (9.5, 301.9) | 76.3 (5.3, 706.2) | 76.9 (5.3, 310) | 82.2 (7, 706.2) | 87.1 (5.3, 706.2) | 74.8 (5.9, 332.6) |

CKD-MBD: chronic kidney disease mineral bone disorder. i-FGF23: intact fibroblast growth factor 23. iPTH: intact parathyroid hormone. TSAT: transferrin saturation. Data were expressed as mean ± SD for normal distributed biomarkers and median (range) for non-normal distributed biomarkers. * means significantly higher value between user and non-user for each medicine.
