# Supplementary material for: High fibroblast growth factor 23 levels are associated with decreased ferritin levels and increased intravenous iron doses in hemodialysis patients
Source: PLoS One. 2017 May 5;12(5):e0176984. doi: 10.1371/journal.pone.0176984 (PMC5419608; doi:10.1371/journal.pone.0176984)
Supplement: S4 Table — (DOCX) [file pone.0176984.s005.docx]

S4 Table. Associations of higher i-FGF23 tertiles with repeated measurements of ferritin and TSAT.

|  | **Log ferritin** | | | | |
| --- | --- | --- | --- | --- | --- |
|  | **F** | **p** | | **F** | **p** |
|  | **Model 1** | | | **Model 2** | |
| Higher intact fibroblast growth factor 23 tertile | 3.1 | | 0.04 | 3.5 | 0.03 |
| Hemoglobin at baseline (g/dL) | 21.7 | | <0.0001 | 21.4 | <0.0001 |
| TSAT at baseline (%) | 131.4 | | <0.0001 | 125.6 | <0.0001 |
| Log i-PTH at baseline | 3.3 | | 0.07 | 5.0 | 0.01 |
| Log 25-hydroxyvitamin D at baseline (ng/mL) | 0.02 | | 0.88 | 0.1 | 0.69 |
| Log1,25-dihydroxyvitamin D at baseline (pg/mL) | 1.5 | | 0.22 | 0.001 | 0.96 |
| Log hs-CRP at baseline | 9.9 | | 0.002 | 10.3 | 0.001 |
|  | **TSAT** | | | | |
|  | **Model 3** | | | **Model 4** | |
| Higher intact fibroblast growth factor 23 tertile | 0.2 | | 0.82 | 0.57 | 0.56 |
| Hemoglobin at baseline (g/dL) | 54.2 | | <0.0001 | 36.9 | <0.0001 |
| Log ferritin at baseline | 137.7 | | <0.0001 | 124.2 | <0.0001 |
| Log i-PTH at baseline | 3.7 | | 0.054 | 6.0 | 0.01 |
| Log 25-hydroxyvitamin D at baseline (ng/mL) | 3.4 | | 0.06 | 4.9 | 0.02 |
| Log 1,25-dihydroxyvitamin D at baseline (pg/mL) | 0.001 | | 0.99 | 1.0 | 0.31 |
| Log hs-CRP at baseline | 13.9 | | 0.0002 | 16.9 | <0.0001 |

TSAT: transferrin saturation, hs-CRP: high sensitive C-reactive protein. Ferritin and TSAT were measured at baseline, 2, 4 and 6 months.

Multivariate repeated measures analysis of ferritin (Model 1) and TSAT (Model 3) were adjusted with age (year), sex (man vs. woman), status of diabetes mellitus (yes vs. no), history of cardiovascular disease (yes vs. no), nutritional state (normal vs. mild to severe malnourished by subjective global assessment), normalized protein catabolic rate (g/kg/day) , hemodialysis vintage (months), dose of intravenous iron supplementation (mg / 6 months), calcium adjusted with albumin (mg/dL), phosphate (mg/dL).

Models 2 and 4 were adjusted with age, sex, status of diabetes mellitus, hemodialysis vintage, dose of intravenous iron supplementation, calcium adjusted with albumin (mg/dL), phosphate (mg/dL), and treatment of vitamin D and phosphate binder (sevelamer and calcium carbonate).
